# Comparison of three microarray probe annotation pipelines: differences in strategies and their effect on downstream analysis

**DOI:** 10.1186/1753-6561-3-S4-S1

**Published:** 2009-07-16

**Authors:** Pieter BT Neerincx, Pierrot Casel, Dennis Prickett, Haisheng Nie, Michael Watson, Jack AM Leunissen, Martien AM Groenen, Christophe Klopp

**Affiliations:** 1 Laboratory of Bioinformatics, Wageningen University and Research centre (WUR), P.O. Box 569, 6700 AN Wageningen, The Netherlands; 2 Sigenae UR875 Biométrie et Intelligence Artificielle/Génétique Cellulaire, Institut National de la Recherche Agrinomique (INRA), BP 52627, 31326 Castanet-Tolosan Cedex, France; 3 Institute for Animal Health (IAH), Compton, nr Newbury, RG20 7NN, UK; 4 Animal Breeding and Genomics Centre, Wageningen University and Research centre (WUR), P.O. Box 338, 6700 AH, Wageningen, The Netherlands

## Abstract

**Background:**

Reliable annotation linking oligonucleotide probes to target genes is essential for functional biological analysis of microarray experiments. We used the IMAD, OligoRAP and sigReannot pipelines to update the annotation for the ARK-Genomics Chicken 20 K array as part of a joined EADGENE/SABRE workshop. In this manuscript we compare their annotation strategies and results. Furthermore, we analyse the effect of differences in updated annotation on functional analysis for an experiment involving *Eimeria *infected chickens and finally we propose guidelines for optimal annotation strategies.

**Results:**

IMAD, OligoRAP and sigReannot update both annotation and estimated target specificity. The 3 pipelines can assign oligos to target specificity categories although with varying degrees of resolution. Target specificity is judged based on the amount and type of oligo versus target-gene alignments (hits), which are determined by filter thresholds that users can adjust based on their experimental conditions. Linking oligos to annotation on the other hand is based on rigid rules, which differ between pipelines.

For 52.7% of the oligos from a subset selected for in depth comparison all pipelines linked to one or more Ensembl genes with consensus on 44.0%. In 31.0% of the cases none of the pipelines could assign an Ensembl gene to an oligo and for the remaining 16.3% the coverage differed between pipelines. Differences in updated annotation were mainly due to different thresholds for hybridisation potential filtering of oligo versus target-gene alignments and different policies for expanding annotation using indirect links. The differences in updated annotation packages had a significant effect on GO term enrichment analysis with consensus on only 67.2% of the enriched terms.

**Conclusion:**

In addition to flexible thresholds to determine target specificity, annotation tools should provide metadata describing the relationships between oligos and the annotation assigned to them. These relationships can then be used to judge the varying degrees of reliability allowing users to fine-tune the balance between reliability and coverage. This is important as it can have a significant effect on functional microarray analysis as exemplified by the lack of consensus on almost one third of the terms found with GO term enrichment analysis based on updated IMAD, OligoRAP or sigReannot annotation.

## Background

High throughput gene expression experiments using microarrays are based on the principle of hybridising strands of nucleotides to form a duplex. For each gene (the target) a microarray contains many copies of one or more short strands (the probes) in small regions on the array called spots. In a microarray experiment expressed sequences or sequences derived thereof are labelled and allowed to hybridise to the probes making the amount of label at each spot an indicator for the amount of gene expression. Since all spots are processed simultaneously, it is essential that all probes have optimal target specificity under the same experimental conditions. Therefore, optimal microarray design requires 1) a completely sequenced reference genome, 2) complete annotation for this reference genome to know what parts may be expressed and 3) complete knowledge about the natural variation amongst the sampled individuals.

Unfortunately there is currently not a single species for which such complete information is available. Although some reference genomes are now close to completion, the recently published first results of the ENCODE project indicate that our knowledge of what is expressed is vastly underestimated [1]. Hence, certainly when a reference genome is not available and array design is primarily based on expressed sequence tags (ESTs), but also for species with a rather complete reference genome, microarray design is sub-optimal. Probe design based on incomplete or erroneous data can lead to serious problems like non-specific probes causing cross hybridisation, orphan probes designed for non-existing targets, missing probes and misleading probes due to erroneous annotation.

Previous re-annotation studies have shown that up to half of the probes for popular microarrays can be problematic as they suffer from cross hybridisation, from detecting something else than what they were designed for, or both [2-7]. The scale of the problem differs for each microarray design and usually reflects differences in probe design criteria and in completeness of the data used for the array design. Erroneous probe to gene assignments at such massive scale were shown to have a dramatic effect on lists of differentially expressed genes [2,6,8] and clustering of genes based on co-expression [6,9]. Surprisingly, Lu and Zang found that it had hardly an effect on sample clustering [6].

Other evidence that current probe annotation is often suboptimal comes from microarray reproducibility studies. Although reproducibility of modern arrays using the same array platform and version is usually good to very good, reproducibility between different array versions even on the same platform can be very poor [10-14]. Re-annotation of the probes using updated data sets and/or using alternative strategies for probe-gene assignment was shown to improve the correlation co-efficient dramatically [10-14]. This suggests that the lack of cross platform reproducibility is mainly caused by poor annotation of probes. Alternative splicing was shown to effect cross platform reproducibility as well as probes from two different vendors might detect the same gene, but not necessarily the same splice variants of that gene [15].

Summarising, it is important to update the annotation for arrays regularly to improve the reliability of probe-target assignments. Three tools to update oligo annotation for microarrays – IMAD, OligoRAP and sigReannot – are described elsewhere in this issue of BMC Proceedings [16-18]. In this manuscript we compare their underlying annotation strategies and the differences in updated annotation for the ARK-Genomics Chicken 20 K array to illustrate the challenges associated with updating annotation and target specificity. We also analyse the effect of the differences in updated annotation on functional analysis of an experiment involving *Eimeria *infected chickens, which was selected as starting material for the joined EADGENE [19] and SABRE [20] workshop on microarray data analysis in November 2008. Finally we propose guidelines for optimal annotation strategies based on the lessons learned from this workshop.

## Methods

### Microarray

The microarray used is the ARK-Genomics Chicken 20 K array consisting of 20.460 probes ranging in length from 60 to 75 nucleotides with the majority of the probes 70 nucleotides long [21]. It was designed in 2005 based on: 1) INSDC (DDBJ/EMBL/GenBank) ESTs/cDNAs including the UMIST ChESTs, 2) Ensembl 30 with gene models based on various sources ranging from highly reliable chicken UniProtKB/Swiss-Prot proteins to relatively unreliable *ab initio in silico *gene predictions, 3) miRBase micro RNAs and 4) a small set of contributed sequences. Microarray data from an experiment with Eimeria infected chickens and using this array was provided as starting material for the EADGENE/SABRE post-analyses workshop [22]. From this experiment a subset of 791 differentially expressed probes based on the MM8-MM24 contrast sample was selected for in depth comparison of the annotation. For the GO term [23] enrichment analysis all 20 K oligos were used.

### Updating annotation

IMAD, OligoRAP and sigReannot were used to update annotation as described elsewhere in this issue [16-18]. At the time of analysis the following database versions were used: SigReannot: Ensembl 50 and UniGene Gga 41; OligoRAP: Ensembl 50, Entrez Gene d.d. 2008-08-26, UniGene Gga 40 and RefSeq 30; IMAD: Ensembl 50, UniGene Gga 39 and DFCI chicken gene indices 11.

Hybridisation filter thresholds were synchronised based on He *et al. *[24] where possible. This means the minimum percentage sequence identity over the complete length of oligo was set to 85% and the minimum length of the longest contiguous stretch was set to 20 nucleotides for OligoRAP and SigReannot or a minimum HSP size of 20 matching nucleotides (not necessarily a contiguous stretch.) OligoRAP's mismatches filter was set to such low values that it was effectively not used.

### GO term enrichment

Different custom array annotation packages based on Ensembl Gene IDs were made using the updated annotation provided by the IMAD, OligoRAP and sigReannot pipelines. These custom annotation packages were made with Bioconductor [25] using the AnnotationDbi package [26]. Three conditions each with a set of up- and a set of down-regulated genes resulted in a total of six gene lists as described elsewhere in this issue [27]. Gene lists were tested for GO term enrichment using a conditional hypergeometric test algorithm from the Bioconductor package GOstats [28]. The significance threshold for this test was set to p values smaller than or equal to 0.05 and only GO terms from the Biological Process (BP) ontology were used.

## Results and discussion

**Figure 1:**
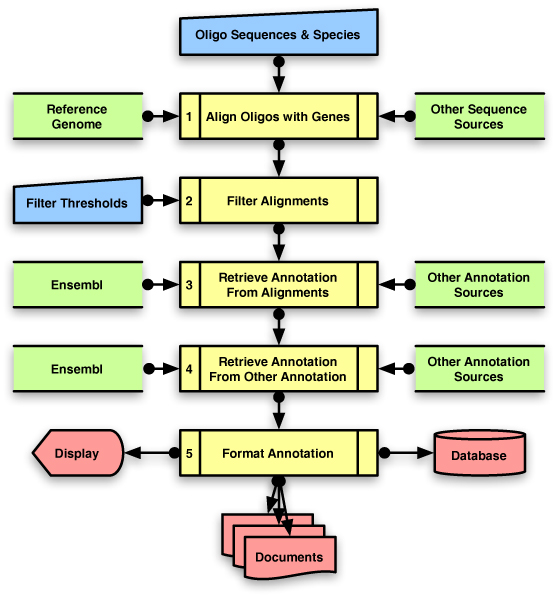
**Consensus Pipeline Overview**. Procedures used by IMAD, OligoRAP and sigReannot to update oligo library annotation can be grouped in 5 steps (yellow), with user inputs (blue), external data sources (green) and results (red).

Figure 1 shows a consensus flowchart with five steps that all three pipelines perform in order to annotate an oligo-nucleotide library.

### Annotation strategies compared

**Figure 2:**
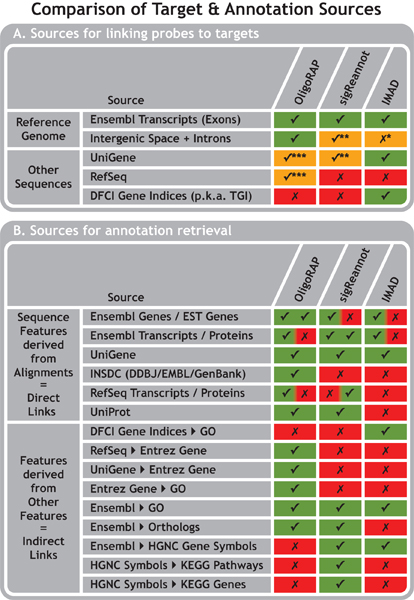
**Comparison of Target and Annotation Sources**. Data sources as used by the corresponding annotation pipelines for linking probes to target genes (A) and for annotation retrieval (B): green checkmark = used, red cross = unused and orange checkmark/cross = partially used. *) IMAD ignores strand information and therefore might contain hits to intergenic space with Ensembl exons annotated on the opposite strand. **) sigReannot can report hits to intergenic space and introns using its UTR/intron extension feature. This requires a hit on a UniGene cluster located in a gene's intron or in a region of 1000 nucleotides up- or downstream of a gene. UTR/intron extension is only performed with sigReannot if there were no hits on Ensembl Genes. OligoRAP on the other hand takes all intergenic space into account irrespective of whether there are hits on Ensembl Genes or not. ***) To prevent redundancy OligoRAP takes only a subset of UniGene and RefSeq accessions into account: those that do not map with high confidence onto the reference assembly.

The first step requires the oligo sequences and species of interest as input and aligns the oligos with potential targets. An overview of datasources used for the alignments is provided in Figure 2A. The primary source of probe targets for all three pipelines is Ensembl [29,30]. OligoRAP uses the entire reference genome assembly for a given species, while IMAD and sigReannot only focus on the parts, which are annotated as Ensembl transcripts. There are two exceptions though.

Firstly, IMAD ignores strand information and hence might link to annotation derived from features located on the opposite strand of a hit. SigReannot is strand-aware, but can link to annotation from the opposite strand if no annotation was found on the hit strand. Most array platforms only detect a single strand and under normal conditions a gene produces only RNA from a single strand. But there can exceptions like in the case of viral reverse transcriptases, some of which can switch templates resulting in chimeric cDNA molecules [31]. IMAD was originally designed for arrays used in experiments involving viral infections, which reflects the design choice to include annotation from both strands. So, depending on array type, sample preparation protocol and/or sample type, linking oligos to genes on the opposite strand can either be a feature or a flaw.

Secondly, sigReannot uses UTR/intron extension in case no hits were found on Ensembl transcripts. The latter means that sigReannot searches UniGene [32] as secondary source for alignments. If a hit is found, the Ensembl API is used to fetch Ensembl genes linked to the found UniGene cluster accessions. For these Ensembl genes the entire sequence including introns is fetched and extended with 1000 nucleotides both up- and downstream. When the oligo can be aligned with these extended sequences, it is linked to the corresponding Ensembl genes. In general Ensembl is relatively conservative in annotating genes and sigReannot's UTR/intron extension is a smart feature to boost the coverage of probes linked to Ensembl gene IDs. On the other hand stretching the boundaries of conservative Ensembl's gene models will also increase the risk of introducing false positive probe to Ensembl gene assignments. Moreover, linking probes to Ensembl gene IDs based on rare splice variants can be misleading. If no signal was observed it means that rare splice variant was not expressed, but that doesn't mean the gene was not expressed at all.

IMAD and OligoRAP also use additional sources for probe-target alignments to increase the coverage of annotated probes. In addition to Ensembl transcripts IMAD aligns probes with UniGene [32] and DFCI Gene Indices (formerly known as TIGR Gene Indices) [33] and OligoRAP aligns with UniGene [32] and RefSeq [32]. The big difference is that IMAD searches the entire databases whereas OligoRAP searches only a sub-set of RefSeq accessions, which are not reliably represented by the reference assembly and a sub-set of UniGene accession neither reliably represented by the reference assembly nor by the RefSeq sub-set. Hence IMAD hits to the different databases can be highly redundant while OligoRAP tries to minimise redundancy.

The second step is to filter the hits based on the quality of the alignments, which relates to the hybridisation potential of a hit. All three pipelines can filter alignments on the percentage of sequence identity. Low quality hits that do not pass this filter, but which do contain small stretches of uninterrupted matches might still contribute to signal on a microarray. Therefore, OligoRAP and sigReannot feature a second filter for the minimum size of what is called the longest contiguous stretch or continuous block, respectively. Finally, OligoRAP has a third filter for the maximum total amount of mismatches. When the probes are not all equally long this filter will produce different results as compared to the percentage identity filter.

In contrast to sigReannot and IMAD, OligoRAP applies the filter step not immediately after aligning oligos with targets, but after all annotation is retrieved instead (after step 4). This allows OligoRAP to check if two or more short hits were derived from intron-separated exons of the same gene. If such hits are found they are merged into a larger hit, which is necessary for OligoRAP, because it aligns with reference genomes as compared to IMAD and sigReannot, which only align with transcripts.

**Figure 3:**
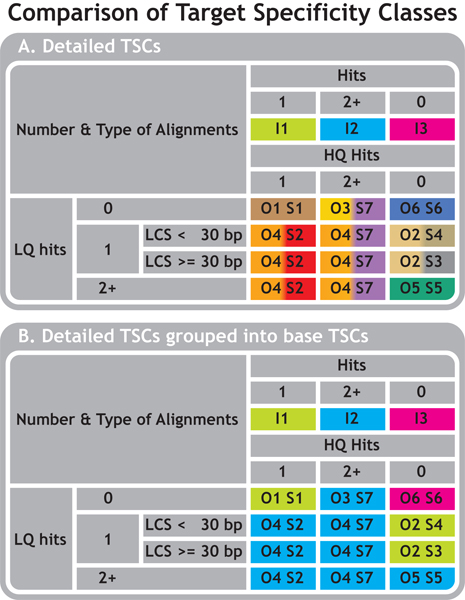
**Comparison of Target Specificity Classes (TSCs)**. Overview of how the TSCs – as defined by the 3 pipelines – (partially) overlap or are divided into smaller sub-categories (A). O = OligoRAP, S = sigReannot, I = IMAD. Numbers indicate the corresponding TSCs. LCS = Longest Contiguous Stretch. IMAD does not differentiate between different hit types. OligoRAP and sigReannot differentiate between High Quality alignments (HQ hits, called "hits" by sigReannot and "primary hits" by OligoRAP) and Low Quality alignments (LQ hits, called "noise" by sigReannot and "secondary hits" by OligoRAP). Figure B shows how more detailed TSCs can be grouped into 3 base TSCs for comparison of the results: one hit (I1 = O1+O2 = S1+S3+S4), multiple hits (I2 = O3+O4+O5 = S2+S5+S7) or no hits at all (I3 = O6 = S6).

Based on the amount and type of hits oligos can be assigned to target specificity classes (TSCs). An overview of how TSCs overlap or differ between the 3 pipelines is given in Figure 3A. IMAD focuses on the big picture the way most biologists are interested in oligo annotation: Are my oligos gene specific or not? This results in three TSCs: gene-specific, non-specific and orphan oligos. OligoRAP and sigReannot on the other hand provide more resolution by differentiating between high quality (HQ) and low quality (LQ) alignments resulting in 7 and 6 TSCs, respectively. OligoRAP uses two thresholds per filter – one for LQ and one for HQ hits – to assign oligos to TSCs. A different approach is used by sigReannot as the percentage sequence identity is used exclusively to filter for HQ hits and the length of the longest contiguous stretch for LQ hits. Figure 3B shows how the more specialised TSCs of OligoRAP and sigReannot can be combined into the more generic TSCs of IMAD for easier comparison of the results produced by IMAD, OligoRAP and sigReannot.

OligoRAP and sigReannot use comparable TSCs in case there was only one HQ hit (TSC O1 & S1), there were multiple LQ hits (TSC O5 & S5), or there were no hits at all (TSC O6 & S6).

When there was only 1 LQ hit, OligoRAP puts these oligos in a single TSC (O2), but sigReannot differentiates between LQ hits with longest contiguous stretches of 30 nucleotides and more (S3) and with stretches of less than 30 nucleotides (S4). The latter TSC contains gene-specific oligos, which are less reliable for detecting lowly expressed genes, because they have the worst signal to noise ratio. By providing an extra TSC for these oligos, users can choose to drop them from further analysis or at least can see quickly they are less reliable. OligoRAP handles this problem differently by allowing users to specify multiple combinations of filter thresholds per run. This allows them to analyse for example the effect of more lenient or stricter thresholds for HQ and/or LQ hits and covers all TSCs instead of just the oligos with only one LQ hit. Analysing different combinations of filter thresholds is also possible with IMAD and sigReannot, but this a bit more work as it requires a user to run the pipeline with the most lenient hybridisation potential filter thresholds followed by post-processing of the results to generate results for more stringent thresholds.

In the case of multiple HQ hits or a mix of HQ and LQ hits sigReannot and OligoRAP classify them differently. SigReannot differentiates between cases with one HQ hit accompanied with one or more LQ hits (TSC S2) and cases with multiple HQ hits with or without LQ hits (TSC S7). OligoRAP on the other hand differentiates between multiple HQ hits (TSC O3) and a mix of HQ and LQ hits (TSC O4). The reason sigReannot differentiates between S2 and S7 while OligoRAP assigns such oligos all to O4 is a difference in annotation retrieval policy (see below). TSC O3 is interesting, because in theory these oligos target shared domains or different highly similar genes. Therefore these oligos could still be informative as such genes are usually involved in similar biological processes just like different splice variants derived from the same gene. In practice however many of the oligos in TSC O3 have multiple HQ hits due to redundancy and this is usually the result of assembly and/or annotation problems. Either way it makes sense to differentiate between these oligos and ones that target a mix of HQ and LQ hits as the latter can suffer from cross-hybridisation with transcripts from highly dissimilar targets and hence are not informative.

Steps three and four consist of annotation retrieval directly from the alignments or indirectly from previously fetched annotation, respectively. There are many differences in annotation features retrieved by the different pipelines, but all pipelines provide links to Ensembl gene IDs, Ensembl Transcripts IDs, UniGene cluster accessions and GO terms IDs derived from Ensembl genes (Figure 2B). The largest differences between the pipelines can be found in the data sources used for annotation expansion using database cross-references. Such indirect links are convenient, but differences here are less important as users can fetch these links themselves from the direct links using other tools like for example BioMart [34]. The annotation present in the final results can depend on the target specificity of an oligo. Both IMAD and sigReannot provide at most only one gene ID/accession per database linked to each oligo. This is accomplished in IMAD by fetching only annotation for the best hit found in each of the databases used for alignment searches. SigReannot on the other hand fetches annotation for the single HQ or LQ hit found (TSC S1, S3 and S4) or for the HQ hit in case it was accompanied by additional LQ hits (TSC S2). Hence, in case there were multiple HQ hits (TSC S7) or multiple LQ hits in absence of HQ hits (TSC S5), sigReannot provides no annotation at all as there is no clear best hit. Although this is an oversimplification it can be an advantage as most downstream analysis tools are limited to accepting only one ID/accession number per oligo and hence that is what most users want. OligoRAP provides all annotation for all hits – both HQ and LQ – it can find. In case there were multiple hits this means the users will have to decide for themselves what hits to take into account. This gives users more control of what annotation to use, but might require additional parsing of OligoRAP annotation.

Finally, the fifth step involves formatting and storing the results in various ways. SigReannot's annotation is provided as collection of tab-delimited flat files. IMAD on the other hand uses a MySQL database to store its results and OligoRAP's native output format is BioMoby [35] XML, but both provide tab-delimited flat files upon request. In addition to data dumps, IMAD provides web-based access to query the updated annotation using a CGI script. SigReannot does not provide web-based access yet, but the data is stored in a BioMart compliant MySQL database with installation of the web-based BioMart front-end planned for a future release. OligoRAP does not provide a web-based interface to query the generated annotation, but the annotation pipeline consists of BioMoby web services allowing users to execute the pipeline remotely themselves instead.

### Effect of differences in annotation strategies on coverage and consensus

**Figure 4:**
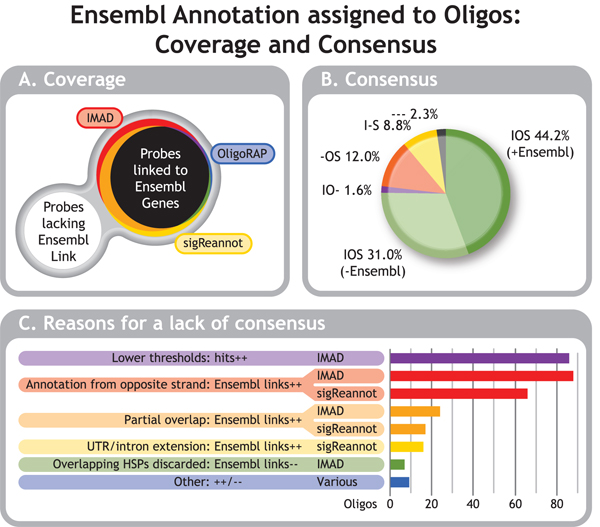
**Ensembl Annotation Assigned to Oligos: Coverage & Consensus**. Venn diagram representing oligos linked to Ensembl gene IDs by the 3 annotation pipelines (A). Colours represent oligos linked to at least one Ensembl gene by all 3 pipelines (417:black), not linked to any Ensembl genes by any of the 3 pipelines (245:white), linked to at least one Ensembl gene only by IMAD (26:red), only by OligoRAP (2:blue), only by sigReannot (13:yellow), by IMAD & OligoRAP (3:purple), OligoRAP & sigReannot (6:green) or by IMAD & sigReannot (79:orange). When an oligo is linked to at least one Ensembl gene by all 3 pipelines this not necessarily means it is linked to the same Ensembl genes, which is depicted as consensus in a pie chart (B). Agreement between all 3 pipelines is subdivided in agreement on the presence or on the absence of links to Ensembl genes. Where only 2 pipelines agree this is not subdivided and hence represents a mix of consensus on presence or absence of annotation. Pipeline's initials indicate the corresponding pipelines share consensus; a dash instead of an initial indicates the corresponding pipelines lack consensus. Reasons for a lack of consensus are sorted by impact (C) and were counted per oligo: ++ = extra hits were found because of this reason, -- = hits were missing because of this reason. If an oligo had multiple hits, multiple reasons can apply, but multiple occurrences of the same reason were counted only once.

A subset of 791 oligos was selected from the experimental data provided for the workshop to assess the effect of the different annotation strategies on coverage. These oligos were selected, because they showed differential gene expression signals. Hence these probes clearly bind transcripts and any orphan oligos in the updated annotation produced by sigReannot, OligoRAP and IMAD indicate false negatives due to incomplete data sources, incomplete annotation strategies or both. The focus for this comparison is on Ensembl gene ID assignments as all three pipelines provide these and hence they can be easily compared. Figure 4A shows a Venn diagram representing the amount of oligos covered with at least one Ensembl gene ID versus probes without any links to Ensembl genes. Slightly more than half (52.7%) of the oligos is linked to at least one Ensembl gene by all three pipelines. Unfortunately with 31.0% the second largest group consists of the oligos, which could not be linked to any Ensembl gene by any pipeline. Although IMAD and OligoRAP can fetch annotation for additional sources to boost annotation coverage, this tends to be less informative, because – apart from assembly gaps – had there been a lot of high quality annotation available for a hit, this would have resulted in an Ensembl gene model. When there was not enough convincing experimental evidence for an Ensembl gene model this often means the hit is only covered by just a few or even a single EST. For the remaining 16.3% of the oligos the coverage differs. The bulk of these (10.0%) are represent oligos linked to Ensembl only by IMAD+sigReannot. Probes linked to Ensembl only by IMAD or only by sigReannot correspond to 3.3% and 1.6%, respectively. Finally, OligoRAP appears to be the most conservative in linking oligos to Ensembl genes as the amounts of oligos linked to Ensembl only by OligoRAP, only by IMAD+OligoRAP and only by OligoRAP+sigReannot are all less than 1%.

In case an oligo was not linked to any Ensembl genes by any of the pipelines, they clearly all agree, but in case two or more pipelines link to Ensembl genes, that does not necessarily mean they link to the same Ensembl genes for the corresponding oligos. Therefore, the consensus between the pipelines in linking oligos to Ensembl genes was determined (Figure 4B). For 44.2% all pipelines link to the same Ensembl gene(s). Together with the 31.0% of the probes without any Ensembl gene link in any of the updated annotation datasets, this means the pipelines agree in approximately three quarters of the cases. In 2.3% of the cases all three pipelines link to Ensembl gene IDs, but these are not (all) the same. Finally, two pipelines agree on either the presence or absence of annotation in 1.9% (IMAD+OligoRAP), 12.1% (OligoRAP+sigReannot) and 8.7% (IMAD+sigReannot) of the cases.

### Reasons for a lack of consensus

In case the annotation pipelines did not agree on the Ensembl genes linked to an oligo the reason for this lack of consensus was determined (Figure 4C). Multiple reasons can apply in case an oligo had multiple links, but if the same reason applied to multiple hits of the same oligo this was counted only once. In 86 cases additional hits were present in the IMAD annotation due to lower thresholds. Although we tried to synchronise hybridisation filter thresholds for this pipeline comparison (see methods), this was not completely possible, because IMAD doesn't have a longest contiguous stretch filter making it more difficult to filter for relevant short hits. To mimic the longest contiguous stretch filter, short stretches (BLAST HSPs) were considered to be positive hits in IMAD when they had a minimum amount of matches equal to the longest contiguous stretch size for OligoRAP and sigReannot. Hence the matches in these short HSPs are not necessarily a contiguous stretch, which explains why some of these hits are missing from OligoRAP and sigReannot. It is questionable whether these extra IMAD hits will be able generate signal in a microarray experiment, but this will depend heavily on the chosen experimental conditions.

In the vast majority of the remaining cases where consensus is lacking, the pipelines initially find the same hits, but judge them differently when deciding whether to link to Ensembl genes based on these hits or not. In 88 and 66 cases oligos are linked to Ensembl genes located on the opposite strand of the hit with IMAD and sigReannot, respectively. The difference is the result of sigReannot not linking to annotation from the opposite strand if there was also annotation on the strand of the hit. Furthermore, sigReannot only takes annotation from the opposite strand into account for HQ hits and hence ignores such annotation for LQ hits. So sigReannot is a bit more conservative in linking to annotation from the opposite strand.

OligoRAP only links a hit to annotation if there is (near) perfect overlap between the hit (oligo – genome alignment) and the annotation (annotated feature – genome alignment). For IMAD and sigReannot this does not apply as they only align the oligos with transcripts. In case a hit extended beyond the borders of a transcript on the genome, IMAD and sigReannot will find a shorter hit covering only the part that overlaps with the transcript. This results in 24 oligos with extra links to Ensembl genes with IMAD and 17 with sigReannot as compared to OligoRAP. The difference between IMAD and sigReannot is the result of partial overlap combined with lower thresholds for IMAD either because of the lack of a contiguous stretch filter in IMAD (2 cases) or because the annotation was derived from the opposite strand and the hit didn't pass sigReannot's HQ hit thresholds (5 cases).

SigReannot's UTR/intron extension feature generates additional links to Ensembl genes for 16 oligos with hits in the vicinity of Ensembl transcripts. IMAD and OligoRAP cannot link to annotation located in the vicinity of a hit on the genome and this explains their absence.

If a BLAST result contains overlapping HSPs these were all ignored by IMAD resulting in 7 oligos where links to Ensembl genes are missing as compared to OligoRAP and sigReannot. Further inspection of these 7 probes revealed they contained repeats and IMAD has been adjusted to include hits from overlapping HSPs.

Finally, the "other" leftover category contains 9 rare cases. In one of these IMAD missed a hit, because it uses BLAST [36,37] with the default low complexity filter switched on, whereas OligoRAP and sigReannot have this switched off. Three oligos had dozens of links to Ensembl, which were only partially shared by the updated annotation sets due to limits on the amount of processed hits. These differences can be considered insignificant, because these oligos were far from target specific and a few hits more or less does not change that. In another 3 cases OligoRAP missed an additional link to Ensembl, because a short alignment was below the detection thresholds for BLAT [38], which is a faster alternative for BLAST and only used by OligoRAP. One of these included a case where OligoRAP's intron gap splicing feature could not help to retrieve the larger alignment with the transcript, because the partial hits were too short. Finally in 2 cases OligoRAP used intron gap splicing to merge shorter hits into longer ones linking to Ensembl genes, which are absent from IMAD and sigReannot annotation. In these cases Ensembl gene models do not support the intron gaps, but in one case additional annotation from ESTs does.

It must be noted that IMAD and sigReannot normally provide maximally a single link to an Ensembl gene per oligo. In case there are multiple hits for an oligo these pipelines will try to find a best one and if this fails not link to Ensembl at all. For this workshop the IMAD and sigReannot teams provided additional data for oligos with multiple hits, so they could all be taken into account and compared, but would users compare standard IMAD and sigReannot data, they might find additional differences due to different prioritisation of hits to find the best one.

In most cases the oligos with multiple hits are non-specific, but further investigation revealed 9 extreme cases of oligos with numerous hits (up to 200) on transcripts representing large gene families or sharing domains such as genes coding for MHC proteins, olfactory proteins, homeobox proteins, protein kinases and potassium voltage-gated channel proteins. Although it was clearly not possible to assign a best hit in these cases linking the oligo to the gene family or shared domain could still be highly informative despite the lower resolution.

### Effect of differences in annotation on GO term enrichment analysis

**Figure 5:**
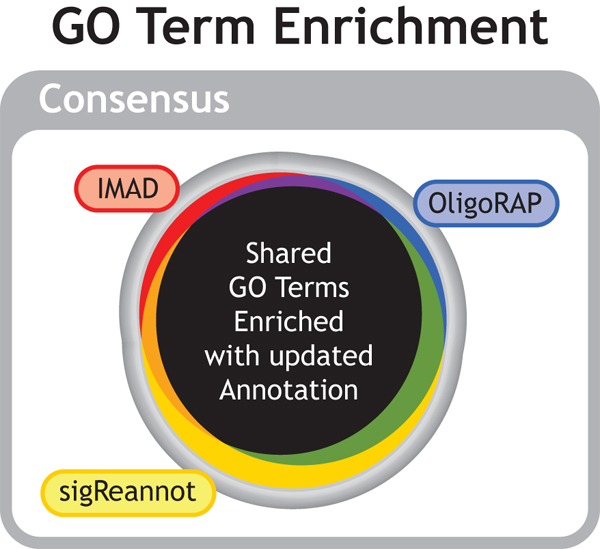
**Consensus on GO Term Enrichment Analysis**. Venn diagram representing consensus on the GO terms retrieved from term enrichment analysis with updated annotation from IMAD, OligoRAP or sigReannot. Colours represent terms found with annotation from all pipelines (172:black), from IMAD only (10:red), from OligoRAP only (8:blue), from sigReannot only (19:yellow), from IMAD & OligoRAP (7:purple), from OligoRAP & sigReannot (26:green) or from IMAD & sigReannot (14:orange).

GO term enrichment analysis was chosen as an example to investigate the results of differences in updated annotation on functional microarray analysis. For this analysis all probes of the ARK-Genomics 20 K chicken array were taken into account, annotation was updated with IMAD, OligoRAP & sigReannot and enrichment of GO terms in the lists of significantly up- or down regulated genes was performed as described by Haisheng *et al. *[27]. Three conditions with each a list of up- and a list of down-regulated genes resulted in a total of six gene lists. For each of these the lists of enriched GO terms derived from the 3 sets of annotation were compared and summarised in a single Venn diagram (Figure 5). With 172 GO terms or 67.2%, the majority of the significantly enriched GO terms (p <= 0.05) were the same in the analysis based on the three updated annotation sets. That also means that there was no consensus on 84 or almost one third of the terms enriched in up- or down-regulated genes. This clearly shows that differences in annotation strategies can have a significant effect on functional analysis.

### Optimal annotation strategies

IMAD only differentiates between oligos with 1 hit, multiple hits or no hits at all and uses a single hybridisation potential filter for sequence identity. This is less advanced than OligoRAP and sigReannot, which differentiate between LQ and HQ hits and introduce a second hybridisation potential filter for short contiguous stretches of matching nucleotides. Despite these differences, basically all three pipelines can divide the oligos into several TSCs giving users an indication of the target specificity of the oligos. Furthermore, depending on experimental conditions, users can customise the parameters for the hybridisation potential filters.

After the pipelines have aligned oligos with potential targets they have to decide whether or not to link to certain annotation based on these alignments and this is where they differ the most. Should the pipelines be very conservative and link only to annotation derived from other sequences that (near) perfectly overlap the alignment of the oligo with the potential target, like OligoRAP does? Or should the annotation strategy be more lenient and include annotation from indirect links like sigReannot does when it uses UTR/intron extension to link indirectly to Ensembl via UniGene? The question basically boils down to whether to prefer reliability over coverage or the other way around. After some discussion during the workshop the biologists present decided they couldn't choose between optimal coverage and optimal reliability. Instead they would prefer to have as much annotation as possible and have metadata attached to the annotation indicating the reliability of the link between the oligo and for example an Ensembl gene. Similar to the target specificity categories one can think of a few annotation link reliability categories that would allow the biologists to filter their results in downstream analysis and see the effect of in- or excluding less reliable annotation in addition to the effect of in- or excluding potential non-specific oligos. We propose the following categories:

1) Direct sequence-based links: annotation was derived from alignment of the oligo with a target sequence.

2) Indirect links

a) Sequence-based and with (near) perfect overlap of the oligo-target alignment with the alignment of the target with the other sequence from which the annotation is derived.

b) Sequence-based and with partial overlap of the oligo-target alignment with the alignment of the target with the other sequence from which the annotation is derived.

c) Sequence-based and without any overlap of the oligo-target alignment with the alignment of the target with the other sequence from which the annotation is derived.

i) Oligo-target alignment is located up- or downstream in the vicinity of the gene from which the annotation is derived.

ii) Oligo-target alignment is located in an intron of the gene from which the annotation is derived.

iii) Oligo-target alignment is located on the opposite strand of the gene from which the annotation is derived.

d) Non sequence-based links. For example in the case of expanding annotation using text mining.

e) Non gene-specific link. For example to a gene family or shared domain.

These categories can be easily expanded where necessary. For category 2 cii one could flag for example whether there was other sequence-based evidence that makes the link more reliable. This would be the case if an oligo aligns with an intron and if there are ESTs that align with both the gene's exons and the intron suggesting the gene model was too conservative and intron retention splice variants do exist.

## Conclusion

Approximately four years after the design of the ARK-Genomics 20 K chicken array almost one third of the probes could no longer or still not be linked to high quality annotation in the form of a link to an Ensembl gene with neither IMAD nor OligoRAP nor sigReannot. This indicates that keeping annotation as well as target specificity up-to-date is important to make most of microarray experiments.

IMAD, OligoRAP and sigReannot can assign oligos to target specificity classes (TSCs) although with different levels of resolution. These TSCs are based on the amount of target each oligo hits and users can specify thresholds for hybridisation potential filter used to determine the impact of these hits. Thereby the hybridisation potential filters combined with the TSCs give users the flexibility to adjust the target specificity estimates to their experimental conditions. In addition it allows them to play safe by discarding potential cross-hybridising probes or live on the edge to get higher annotation coverage. In contrast to target specificity users have no control over the annotation that is fetched based on the hits of the oligos with potential targets. Fetching annotation from indirect relationships between oligos and potential targets can help to boost coverage, but will also result in varying levels of reliability of the updated annotation. Not only have users currently no control over which annotation is retrieved, they currently also cannot see the difference between annotation from more reliable direct links and from less reliable indirect links. Based on the feedback from the EADGENE/SABRE post-analysis workshop we therefore suggest annotation link reliability categories be added to indicate the type of relationship between oligos and their annotation. Adding such indicators for the reliability of the annotation will be an important step in the future development of IMAD, OligoRAP and sigReannot and allow users to fine tune the balance between reliability and coverage. This is important as it can have a significant effect on functional analysis of microarray data as exemplified by the lack of consensus on almost one third of the terms found with GO term enrichment analysis using updated annotation generated with IMAD, OligoRAP and sigReannot.

## Further information and supplemental data

Links to supplemental files with annotation as used for the workshop as well as presentations as presented at the workshop are available from the EADGENE portal:



## Competing interests

The authors declare that they have no competing interests.

## Authors' contributions

PBTN generated OligoRAP annotation, PC generated sigReannot annotation and MW generated IMAD annotation. PBTN, PC, MW, DP & CK analysed the generated annotation and compared the different annotation strategies. HN studied the effect of different pipelines and different filter thresholds on GO term over-representation analysis. JAML, MAMG, MW and CK secured funding and managed the project. PBTN drafted the manuscript, which was improved with the help of all other authors. All authors read and approved the final manuscript.

## References

[B1] Birney E, Stamatoyannopoulos JA, Dutta A, Guigo R, Gingeras TR, Margulies EH, Weng Z (2007). Identification and analysis of functional elements in 1% of the human genome by the ENCODE pilot project. Nature.

[B2] Gautier L, Moller M, Friis-Hansen L, Knudsen S (2004). Alternative mapping of probes to genes for Affymetrix chips. BMC Bioinformatics.

[B3] Harbig J, Sprinkle R, Enkemann SA (2005). A sequence-based identification of the genes detected by probesets on the Affymetrix U133 plus 2.0 array. Nucleic Acids Res.

[B4] Dai M, Wang P, Boyd AD, Kostov G, Athey B, Jones EG, Bunney WE, Myers RM, Speed TP, Akil H, Watson SJ, Meng F (2005). Evolving gene/transcript definitions significantly alter the interpretation of GeneChip data. Nucleic Acids Res.

[B5] Perez-Iratxeta C, Andrade MA (2005). Inconsistencies over time in 5% of NetAffx probe-to-gene annotations. BMC Bioinformatics.

[B6] Lu X, Zhang X (2006). The effect of GeneChip gene definitions on the microarray study of cancers. Bioessays.

[B7] Orlov YL, Zhou J, Lipovich L, Shahab A, Kuznetsov VA (2007). Quality assessment of the Affymetrix U133A&B probesets by target sequence mapping and expression data analysis. In Silico Biol.

[B8] Lu J, Lee JC, Salit ML, Cam MC (2007). Transcript-based redefinition of grouped oligonucleotide probe sets using AceView: high-resolution annotation for microarrays. BMC Bioinformatics.

[B9] Okoniewski MJ, Miller CJ (2006). Hybridization interactions between probesets in short oligo microarrays lead to spurious correlations. BMC Bioinformatics.

[B10] Hwang KB, Kong SW, Greenberg SA, Park PJ (2004). Combining gene expression data from different generations of oligonucleotide arrays. BMC Bioinformatics.

[B11] Elo LL, Lahti L, Skottman H, Kylaniemi M, Lahesmaa R, Aittokallio T (2005). Integrating probe-level expression changes across generations of Affymetrix arrays. Nucleic Acids Res.

[B12] Mecham BH, Klus GT, Strovel J, Augustus M, Byrne D, Bozso P, Wetmore DZ, Mariani TJ, Kohane IS, Szallasi Z (2004). Sequence-matched probes produce increased cross-platform consistency and more reproducible biological results in microarray-based gene expression measurements. Nucleic Acids Res.

[B13] Carter SL, Eklund AC, Mecham BH, Kohane IS, Szallasi Z (2005). Redefinition of Affymetrix probe sets by sequence overlap with cDNA microarray probes reduces cross-platform inconsistencies in cancer-associated gene expression measurements. BMC Bioinformatics.

[B14] Mecham BH, Wetmore DZ, Szallasi Z, Sadovsky Y, Kohane I, Mariani TJ (2004). Increased measurement accuracy for sequence-verified microarray probes. Physiol Genomics.

[B15] Lee JC, Stiles D, Lu J, Cam MC (2007). A detailed transcript-level probe annotation reveals alternative splicing based microarray platform differences. BMC Genomics.

[B16] Prickett D, Watson M (2009). IMAD: Flexible annotation of microarray sequences. BMC Proceedings.

[B17] Neerincx PBT, Rauwerda H, Nie H, Groenen MAM, Breit TM, Leunissen JAM (2009). OligoRAP – An Oligo Re-Annotation Pipeline to improve annotation and estimate target specificity. BMC Proceedings.

[B18] Casel P, Moreews F, Lagarrigue S, Klopp C (2009). sigReannot: an oligo-set re-annotation pipeline based on similarities with the Ensembl transcripts and Unigene clusters. BMC Proceedings.

[B19] European Animal Disease Genomic Network of Excellence (EADGENE). http://www.eadgene.info/.

[B20] Cutting Edge Genomics for Sustainable Animal Breeding (SABRE). http://www.sabre-eu.eu/.

[B21] ARK-Genomics Chicken 20 K Oligo Array. http://www.arkgenomics.org/microarrays/.

[B22] Hedegaard J, Bicciato S, Bonnet A, Boo MR, Buitenhuis B, Collado-Romero M, Conley LN (2009). Methods for interpreting lists of affected genes obtained in a DNA microarray experiment. BMC Proceedings.

[B23] Ashburner M, Ball CA, Blake JA, Botstein D, Butler H, Cherry JM, Davis AP, Dolinski K, Dwight SS, Eppig JT, Harris MA, Hill DP, Issel-Tarver L, Kasarskis A, Lewis S, Matese JC, Richardson JE, Ringwald M, Rubin GM, Sherlock G (2000). Gene ontology: tool for the unification of biology. The Gene Ontology Consortium. Nat Genet.

[B24] He Z, Wu L, Li X, Fields MW, Zhou J (2005). Empirical establishment of oligonucleotide probe design criteria. Appl Environ Microbiol.

[B25] Gentleman RC, Carey VJ, Bates DM, Bolstad B, Dettling M, Dudoit S, Ellis B, Gautier L, Ge Y, Gentry J, Hornik K, Hothorn T, Huber W, Iacus S, Irizarry R, Leisch F, Li C, Maechler M, Rossini AJ, Sawitzki G, Smith C, Smyth G, Tierney L, Yang JY, Zhang J (2004). Bioconductor: open software development for computational biology and bioinformatics. Genome Biol.

[B26] Bioconductor – open source software for Bioinformatics. http://www.bioconductor.org.

[B27] Nie H, Neerincx PBT, Poel Jvd, Ferrari F, Bicciato S, Leunissen JAM, Groenen MAM (2009). Microarray data mining using Bioconductor packages. BMC Proceedings.

[B28] Falcon S, Gentleman R (2007). Using GOstats to test gene lists for GO term association. Bioinformatics.

[B29] Flicek P, Aken BL, Beal K, Ballester B, Caccamo M, Chen Y, Clarke L (2008). Ensembl 2008. Nucleic Acids Res.

[B30] Stabenau A, McVicker G, Melsopp C, Proctor G, Clamp M, Birney E (2004). The Ensembl core software libraries. Genome Res.

[B31] Ouhammouch M, Brody EN (1992). Temperature-dependent template switching during in vitro cDNA synthesis by the AMV-reverse transcriptase. Nucleic Acids Res.

[B32] Wheeler DL, Barrett T, Benson DA, Bryant SH, Canese K, Chetvernin V, Church DM (2008). Database resources of the National Center for Biotechnology Information. Nucleic Acids Res.

[B33] Quackenbush J, Liang F, Holt I, Pertea G, Upton J (2000). The TIGR gene indices: reconstruction and representation of expressed gene sequences. Nucleic Acids Res.

[B34] Smedley D, Haider S, Ballester B, Holland R, London D, Thorisson G, Kasprzyk A (2009). BioMart – biological queries made easy. BMC Genomics.

[B35] Wilkinson MD, Senger M, Kawas E, Bruskiewich R, Gouzy J, Noirot C, Bardou P (2008). Interoperability with Moby 1.0 – it's better than sharing your toothbrush!. Brief Bioinform.

[B36] Altschul SF, Gish W, Miller W, Myers EW, Lipman DJ (1990). Basic local alignment search tool. J Mol Biol.

[B37] Altschul SF, Madden TL, Schaffer AA, Zhang J, Zhang Z, Miller W, Lipman DJ (1997). Gapped BLAST and PSI-BLAST: a new generation of protein database search programs. Nucleic Acids Res.

[B38] Kent WJ (2002). BLAT – the BLAST-like alignment tool. Genome Res.

